# Adding Far-Red to Red-Blue Light-Emitting Diode Light Promotes Yield of Lettuce at Different Planting Densities

**DOI:** 10.3389/fpls.2020.609977

**Published:** 2021-01-15

**Authors:** Wenqing Jin, Jorge Leigh Urbina, Ep Heuvelink, Leo F. M. Marcelis

**Affiliations:** ^1^Horticulture and Product Physiology, Department of Plant Sciences, Wageningen University and Research, Wageningen, Netherlands; ^2^Priva, De Lier, Netherlands

**Keywords:** vertical farm, LED, far-red, lettuce, light use efficiency, yield component analysis

## Abstract

The economic viability and energy use of vertical farms strongly depend on the efficiency of the use of light. Increasing far-red radiation (FR, 700–800 nm) relative to photosynthetically active radiation (PAR, 400–700 nm) may induce shade avoidance responses including stem elongation and leaf expansion, which would benefit light interception, and FR might even be photosynthetically active when used in combination with PAR. The aims of this study are to investigate the interaction between FR and planting density and to quantify the underlying components of the FR effects on growth. Lettuce (*Lactuca sativa* cv. Expertise RZ) was grown in a climate chamber under two FR treatments (0 or 52 μmol m^–2^ s^–1^) and three planting densities (23, 37, and 51 plants m^–2^). PAR of 89% red and 11% blue was kept at 218 μmol m^–2^ s^–1^. Adding FR increased plant dry weight after 4 weeks by 46–77% (largest effect at lowest planting density) and leaf area by 58–75% (largest effect at middle planting density). Radiation use efficiency (RUE: plant dry weight per unit of incident radiation, 400–800 nm) increased by 17–42% and incident light use efficiency (LUE_inc_: plant dry weight per unit of incident PAR, 400–700 nm) increased by 46–77% by adding FR; the largest FR effects were observed at the lowest planting density. Intercepted light use efficiency (LUE_int_: plant dry weight per unit of intercepted PAR) increased by adding FR (8–23%). Neither specific leaf area nor net leaf photosynthetic rate was influenced by FR. We conclude that supplemental FR increased plant biomass production mainly by faster leaf area expansion, which increased light interception. The effects of FR on plant dry weight are stronger at low than at high planting density. Additionally, an increased LUE_int_ may contribute to the increased biomass production.

## Introduction

Vertical farming is a relatively new plant production system, where plants are grown without solar light in many layers above each other. Plants receive light from lamps (usually light-emitting diodes, LEDs) and all growth conditions can be fully controlled. This production system scores high on sustainability since crops can be grown without the use of pesticides, without nutrient emission, and with high water and land use efficiencies (SharathKumar et al., 2020). However, the energy consumption is high, especially for lighting. Therefore, there is an urgent need for increased light use efficiency.

Light use efficiency (LUE) can be defined in several ways. For overall performance of vertical farming, the fresh yield of harvested product per unit of emitted light by light source is the most relevant definition. The efficiency of the lighting may also refer to the ratio between plant dry weight and total photon flux incident on the canopy, which is called radiation use efficiency (RUE, g mol^–1^), or the ratio between plant dry weight and total photosynthetic photon flux intercepted by the canopy, which is called intercepted light use efficiency (LUE_int_, g mol^–1^). RUE is directly connected to the energy use efficiency (Pennisi et al., 2020) and LUE_int_ indicates the efficiency of the plants transforming intercepted photons into biomass.

Far-red radiation (FR, 700–800 nm) is relatively little absorbed by leaves and mostly reflected or transmitted (Taiz et al., 2015). In nature where the sun is the sole light source, the ratio between red (R) and FR (R/FR ratio) perceived by leaves decreases when vegetation proximity or shading by leaves occurs. R/FR ratio determines the equilibrium of Pfr and Pr in plant (Pierik and De Wit, 2014). Pr and Pfr are two photo-convertible isomers of phytochrome, which could transform to each other by absorbing R or FR (Demotes-Mainard et al., 2016). A rebalanced equilibrium by lowered R/FR ratio induces shade avoidance syndrome (SAS), which includes responses such as increased stem length and/or leaf elongation, leaf moving upward (hyponasty), a higher fraction of assimilate partitioning to stem, and/or increased specific leaf area (Franklin, 2008; Vos et al., 2010; Bongers et al., 2014).

As the application of light-emitting diodes (LEDs) expanded in the past decade, several studies on FR have been conducted for further understanding its effect on crop growth. Park and Runkle (2017) reported 28–50% shoot dry weight increase by adding 16–64 μmol m^–2^ s^–1^ FR on top of 128 μmol m^–2^ s^–1^ R, and 32 μmol m^–2^ s^–1^ blue in geranium and snapdragon. Zou et al. (2019) observed a 49% leaf area increase and 39% biomass production increase by addition of 50 μmol m^–2^ s^–1^ FR during the whole photoperiod in lettuce with the background 200 μmol m^–2^ s^–1^ R and B (*R*/*B* = 7:1). Thus, adding FR is a possible approach to increase plant light interception and biomass production.

Planting density affects R/FR ratio as well, since R will be mostly absorbed by plants but FR only to a small extent. A lowered R/FR ratio will be perceived by plants in a higher planting density. In addition, adding FR to photosynthetically active radiation (PAR, 400–700 nm) may increase the efficiency of photosystem II electron transport and thus increase the net instantaneous photosynthesis rate (Zhen and van Iersel, 2017; Zou et al., 2019). Some authors even proposed to consider a part of FR (700–750 nm) as PAR (Zhen and Bugbee, 2020) when it is applied in combination with PAR such as R and B, although some others did not find an increment in instantaneous net photosynthesis rate when plants acclimated to FR-enriched light were compared with plants under light without FR (Ji et al., 2019; Zhang et al., 2019). Although several studies on the effect of FR on lettuce growth have been conducted (Meng and Runkle, 2019; Zou et al., 2019), a study quantifying the contribution of underlying components on FR improved crop growth is lacking.

Yield component analysis has been used to quantify contributions of underlying components of yield in several studies (Higashide and Heuvelink, 2009; Li et al., 2014; Ji et al., 2019). The aims of this study are to investigate the interaction between FR and planting density and to quantify the underlying components of the FR effects on growth. We hypothesize that FR addition increases the partitioning to the shoot, resulting in an increased biomass production by enlarged leaf area and hence light interception. We expected that the effects on light interception are in particular of importance when plants are widely spaced. For testing this hypothesis, a climate room experiment was conducted with lettuce applying two levels of FR at three planting densities.

## Materials and Methods

### Plant Material and Experimental Setup

Lettuce (*Lactuca sativa* cv. Expertise RZ) was grown in a climate room with six compartments divided by white plastic screens (treatment distribution see Supplementary Table S1). Seeds were sown in 108-cell plug trays filled with a mix of peat and perlite (Lentse Potgrond, Horticoop, Netherlands). Germination procedure involved 2 days in dark followed by 5 days in light at 18 h light/6 h dark with a light intensity of 132 ± 1.5 μmol m^–2^ s^–1^ provided by red (R) and blue (B) LEDs (89% R and 11% B) (GreenPower LED production module, 2nd generation, Philips). Seven days after sowing, seedlings with two cotyledons were transplanted to individual pots (9 × 9 × 10 cm, L × W × H) filled by expanded clay grid (4–8 mm; Jongkind hydrocorns, Netherlands) and were grown for 28 days. Light and planting density treatments started at the same time. Pots were always in 1.5–2.0 cm layer of nutrient solution. Nutrient solution [electrical conductivity (EC) 2.3 dS m^–1^ and pH 5.8], containing 0.38 mM NH_4_^+^, 8.82 mM K^+^, 4.22 mM Ca^2+^, 1.15 mM Mg^2+^, 12.92 mM NO_3_^–^, 1.53 mM Cl^–^, 1.53 mM SO_4_^2–^, 0.12 mM HCO_3_^–^, 1.53 mM H_2_PO_4_^–^, 0.38 mM SiO_3_^2–^, 30.67 μM Fe^3+^, 3.83 μM Mn^2+^, 3.83 μM Zn^2+^, 38.33 μM B, 0.77 μM Cu^2+^, and 0.38 μM Mo, was applied from the second day after transplanting. Nutrient solution was completely renewed twice a week to keep EC, composition, and pH stable. During the whole cultivating period, temperature and relative humidity (RH) were maintained at 22 ± 0.0°C and 75 ± 0.1% for photoperiod and 20 ± 0.0°C and 79 ± 0.2% for dark period, respectively. CO_2_ concentration was kept at 752 ± 6.2 ppm. These data are average with standard errors of means of three blocks (replications in time).

### Light and Planting Density Treatments

**FIGURE 1:**
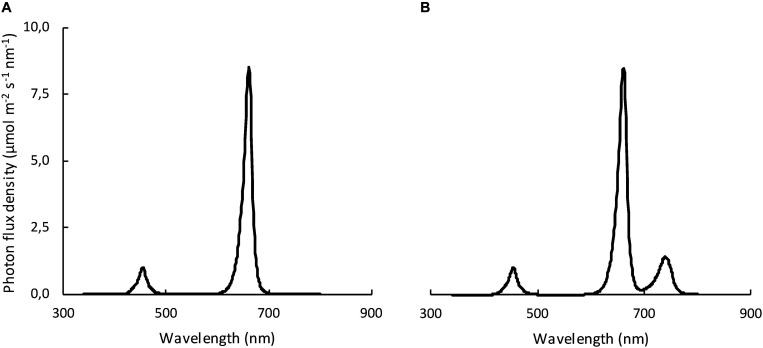
Spectral distribution of the two light treatments: **(A)** without far-red (RB); **(B)** with far-red (RB + FR). Spectra were recorded and averaged on 21 locations along the cultivated area at pot level, measured by a spectroradiometer.

Two far-red (FR) treatments (with FR and without FR: RB + FR and RB, respectively) in combination with three planting densities [23 (low), 37 (middle), and 51 (high) plants m^–2^] were applied. PAR was 218 ± 0.5 μmol m^–2^ s^–1^ and 219 ± 1.5 μmol m^–2^ s^–1^ (89% R and 11% B, GreenPower LED production module, 2nd generation, Philips) for treatment with and without FR, respectively. In the treatment with FR, the FR intensity (700–800 nm) was 52 ± 0.2 μmol m^–2^ s^–1^ provided by GreenPower LED production module, Philips (Figure 1). These intensities of R, B, and FR resulted in phytochrome stationary state (PSS) of 0.83 (RB + FR) and 0.88 (RB) as calculated by the procedure of Sager et al. (1988). The choice for light intensity, photoperiod, and red/blue ratio of the light was based on what is commonly used in vertical farms. The FR level was chosen such that a distinct effect on plant growth could be expected, but not so high that it would never be realistic for a vertical farm. Light measurements were done at pot height using a quantum sensor (LI-COR, LI-250A Lincoln, United States) and with a spectroradiometer (Apogee Instruments model SS-110, Utah, United States). In each of the three blocks, the light intensity was measured at 24 locations per plot. The presented average values and their standard errors were based on three blocks per treatment.

Plants were distributed equidistantly following a chess board pattern. The outer row of plants in each plot was considered as border plants and not used for measurements. After each destructive harvest, plants were relocated to keep the original planting density.

### Biomass and Leaf Net Photosynthesis Rate

Destructive measurements were conducted at 0, 7, 14, 21, and 28 days after transplanting (DAT). Individual plant pictures from the top were taken before destructive measurement for estimation of projected leaf area (PLA) at 14, 21, and 28 DAT. Leaf area was measured by a leaf area meter (LI-3100 Area Meter, LI-COR, Lincoln, United Kingdom). Fresh and dry weights (forced air oven at 105°C for 24 h) of shoot and root were determined. As the stem of this cultivar was extremely small, leaf dry weight was considered to be equal to the shoot dry weight.

At 20 DAT, leaf net photosynthesis rate was measured with a portable gas exchange system (LI-6400; LI-COR, Lincoln, United Kingdom) using a transparent cuvette under the following growing conditions: incident light intensity: 220 μmol m^–2^ s^–1^ with 90% R and 10% B, 22°C for the temperature, 75% for relative humidity, and CO_2_ concentration for 700 ppm. Measurements were performed on fully expanded and unshaded leaves.

### Light Interception and Use Efficiency of Light

Floor coverage fraction was calculated based on individual plant projected leaf area and planting density. Daily floor coverage fraction was calculated by linear interpolation between measurement days at 14, 21, and 28 DAT. Floor coverage fraction at 0 DAT was assumed to be zero. Daily light interception was calculated as the product of incident light intensity and floor coverage fraction at that day. For these calculations, the incident light intensity was measured before start of the experiment at half the final height of the plants. Considering the small height of the lettuce plants, this is a reasonable estimate of the average light intensity.

Radiation use efficiency (RUE) was calculated by dividing plant total dry weight by the cumulative incident radiation, including PAR and FR (400–800 nm), at canopy top level. Incident light use efficiency (LUE_inc_) was calculated as the ratio between plant total dry weight and cumulative incident PAR (400–700 nm). Intercepted light use efficiency (LUE_int_) was calculated as the ratio between plant total dry weight and cumulative intercepted PAR.

### Yield Component Analysis

**FIGURE 2:**
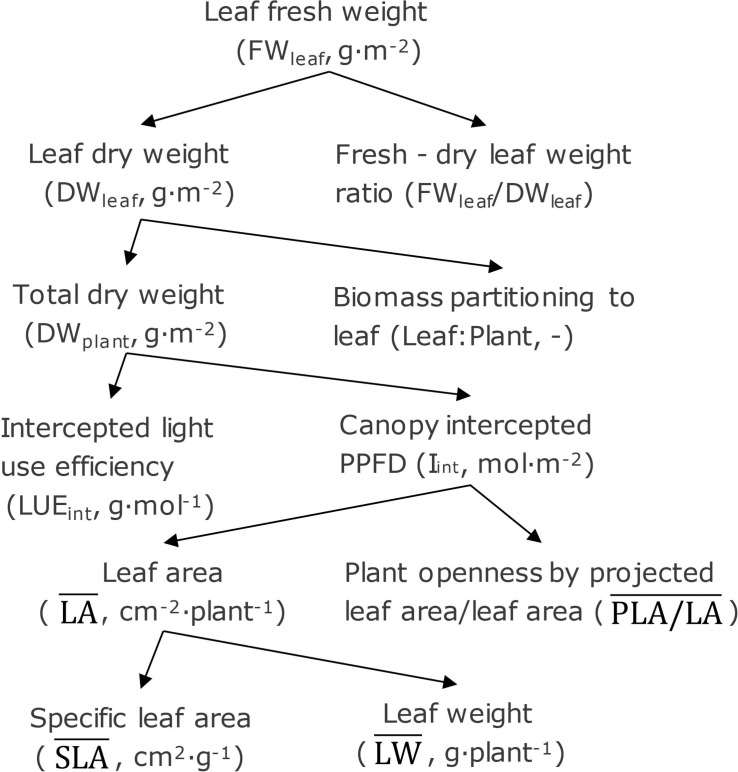
Leaf fresh weight separated into underlying components. Abbreviation and unit are given in between brackets.

Treatment effects can be analyzed by breaking down fresh weight into underlying components (Figure 2). In this analysis, leaf fresh weight (FW_leaf_) is the product of leaf dry weight (DW_leaf_) and the fresh/dry leaf weight ratio (FW_leaf_/DW_leaf_). Leaf dry weight is the product of total dry weight (DW_plant_) and fraction of biomass partitioning to leaf (leaf/plant). Canopy-intercepted photosynthetic photon flux density (PPFD) (I_int_), which is the cumulative PPFD interception during the whole cultivating period (0–28 DAT), and the dry weight production per unit intercepted PPFD (LUE_int_) determine the total dry weight. Canopy-intercepted PPFD was calculated based on projected leaf area, which is determined by leaf area per plant ($\bar{\text{LA}}$) and plant openness defined as the ratio between projected leaf area and leaf area ($\bar{\mathrm{PLA}/\mathrm{LA}}$). Leaf dry weight ($\bar{\text{LW}}$) and specific leaf area ($\bar{\text{SLA}}$) determine the leaf area. The $\bar{\text{LA}}$, $\bar{\mathrm{PLA}/\mathrm{LA}}$, $\bar{\text{SLA}}$, and $\bar{\text{LW}}$ were averaged over 14, 21, and 28 DAT representing the average levels of all parameters during the whole cultivating period (0–28 DAT).

### Statistical Setup and Analysis

A randomized complete block design was applied. The experiment was repeated three times, with repetitions in time representing three blocks (*n* = 3). At 28 DAT for high planting density and no additional FR, only data from two blocks were used. The third block gave an extreme outlier for leaf/root ratio, 15 instead of 4–6; therefore, these measurements were not included in the final analysis. There were four–six replicate plants per block for each destructive measurement and three for photosynthesis. For each block, a new randomization of the light treatment positions was done. Analysis of variance was used to determine treatment effects using Genstat software (18th edition, United Kingdom). Normality of the residuals was tested using the Shapiro–Wilk test, and equal variances were assumed as this could not be tested with only three repetitions. Mean separation was done with Fisher’s protected least significant difference (LSD) test (*P* < 0.05 or *P* < 0.10). In each repetition, the measurements were based on three–six replicate plants, as indicated in the description of the measurements.

FR effects were tested for each planting density separately using a one-way ANOVA in component analysis. Since for such a test the total number of experimental units was only six, a level of significance of 0.10 was applied as is normal in such cases (Ott and Longnecker, 2010). FR effects were also tested together with planting density using a two-way ANOVA in other figures and results with the level of significance of 0.05.

## Results

### Biomass, Leaf Area, Leaf/Root Ratio, Intercepted PPFD, LUE_inc_, LUE_int_, RUE, and SLA

**FIGURE 3:**
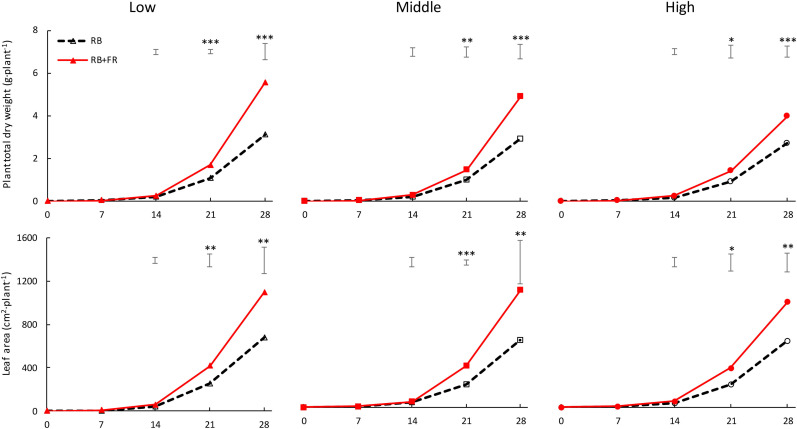
Time course of total dry weight of lettuce plants (g plant^–1^, upper layer) and plant leaf area (cm^2^ plant^–1^, lower layer) when grown without (RB) or with (RB + FR) 52 μmol m^–2^ s^–1^ far-red radiation (FR) intensity, at three planting densities (low, middle, and high being 23, 37, and 51 plants m^–2^, respectively). Solid lines represent RB + FR treatment and dashed lines indicate RB treatment. Bars on top of each day represent least significant difference. Significant effect of FR: ^∗^*P* < 0.10, ^∗∗^*P* < 0.05, and ^∗∗∗^*P* < 0.01 Data are means of three blocks (*n* = 3) each with four–six replicate plants.

At all three planting densities, plant dry weight and leaf area were higher when FR was added (Figure 3). Neither plant dry weight nor leaf area per plant was affected by planting density when no FR was present. Dry weight per plant in the presence of FR was lower at higher planting density. The effects of FR on plant dry weight and leaf area were smaller at higher planting density. Adding FR increased plant dry weight after 4 weeks by 46–77% (largest effect at lowest planting density) and leaf area by 58–75% (largest effect at middle planting density).

**FIGURE 4:**
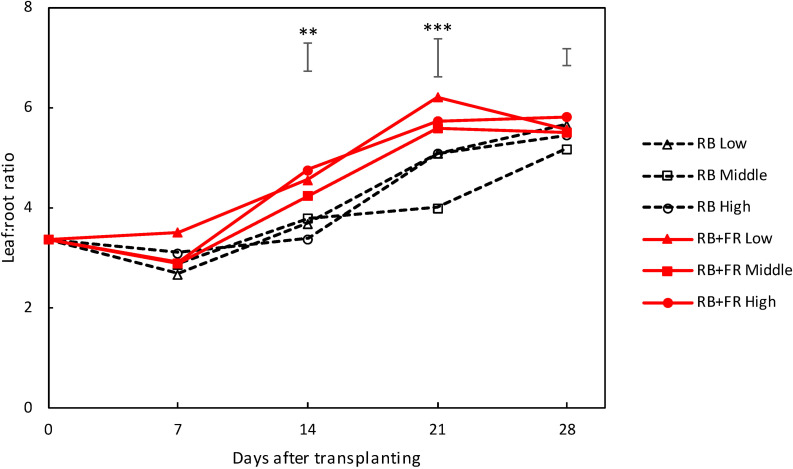
Pattern of leaf/root ratio over time for lettuce plants grown with or without far-red radiation (FR) at three planting densities (low, middle, and high being 23, 37, and 51 plants m^–2^, respectively). Solid lines represent RB + FR treatment and dashed lines indicate RB treatment. Bars on top of each day represent least significant difference. Significant FR effect: ^∗∗^*P* < 0.05 and ^∗∗∗^*P* < 0.01. Data are means of two (*n* = 2) or three blocks (*n* = 3) each with four–six replicate plants.

Leaf/root ratio increased during plant development. FR increased leaf/root ratio significantly at 14 and 21 DAT (Figure 4). Planting density did not significantly affect leaf/root ratio.

**FIGURE 5:**
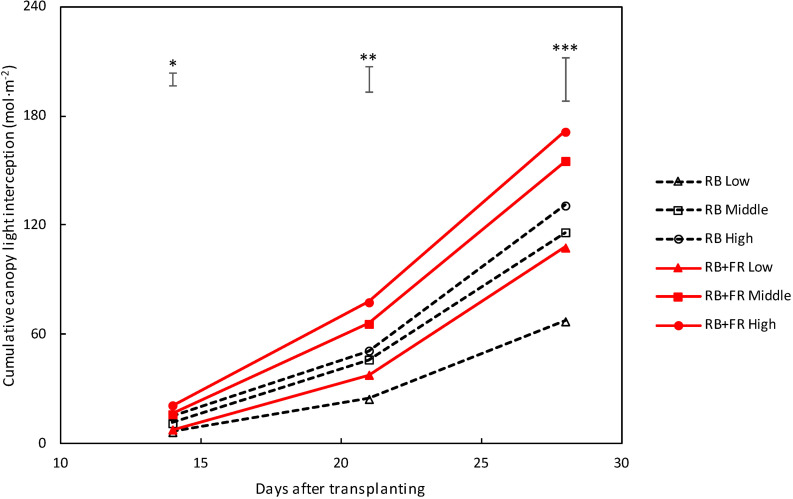
Intercepted photosynthetic photon flux density (PPFD) of lettuce canopy grown at three planting densities (low, middle, and high being 23, 37, and 51 plants m^–2^). Solid lines represent with far-red radiation (FR) treatment (52 μmol m^–2^ s^–1^ FR) and dashed lines indicate treatment without FR. Light was cumulated from 14 to 28 DAT. Bars on top of each day represent least significant difference. Significant FR effect: ^∗^*P* < 0.10, ^∗∗^*P* < 0.05 and, ^∗∗∗^*P* < 0.01. Data are means of two blocks (*n* = 2) each with four–six replicate plants.

Canopy-intercepted PPFD increased with time (Figure 5), which was related to the increase in leaf area. Intercepted PPFD was larger for plants grown with FR compared to plants grown without FR, at all three planting densities.

**FIGURE 6:**
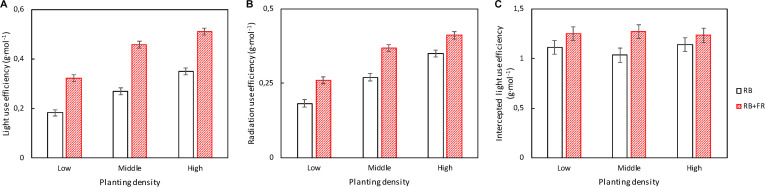
**(A)** Incident light use efficiency [LUE_inc_, which is plant dry weight per unit of incident photosynthetically active radiation (PAR)]; **(B)** radiation use efficiency [RUE, which is plant dry weight per unit of incident radiation including PAR and far-red radiation (FR)]; and **(C)** intercepted light use efficiency [LUE_int_, which is the plant dry weight per unit of canopy-intercepted photosynthetic photon flux density (PPFD)] of lettuce plants grown at three planting densities (23, 37, and 51 plants m^–2^), with (52 μmol m^–2^ s^–1^) and without FR at 28 days after transplanting (DAT). Error bars indicate standard errors of means. None of these three parameters showed a significant interaction between FR and planting density (*P* > 0.25). For incident light use efficiency **(A)** and radiation use efficiency **(B)**, effects of both FR [least significant difference (LSD) = 0.024 and LSD = 0.022, respectively, *n* = 3] and planting density (LSD = 0.030 and LSD = 0.027, respectively, *n* = 2) were significant (*P* < 0.001). For intercepted light use efficiency **(C)**, planting density effect was not significant (*P* = 0.87) and FR effect was significant (*P* = 0.043; LSD = 0.15; *n* = 2). Data are means of two (*n* = 2) or three blocks (*n* = 3) each with four–six replicate plants.

FR significantly increased incident light use efficiency (LUE_inc_, Figure 6A) and radiation use efficiency (RUE, Figure 6B) at all three planting densities. Radiation use efficiency (RUE: plant dry weight per unit of incident radiation, 400–800 nm) increased by 17–42% and incident light use efficiency (LUE_inc_: plant dry weight per unit of incident PAR, 400–700 nm) increased by 46–77% by FR; the largest FR effects were observed at the lowest planting density. Intercepted light use efficiency (LUE_int_: plant dry weight per unit of intercepted PAR) also increased by FR, but to a lesser extent (8–23%) (Figure 6C).

**FIGURE 7:**
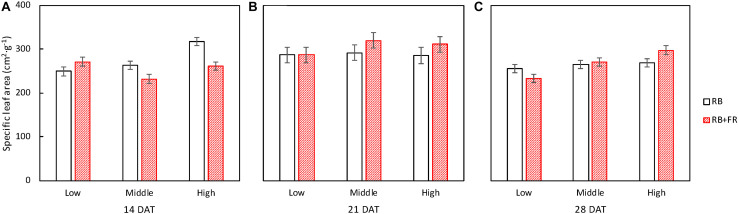
Specific leaf area (SLA) of lettuce plants grown at three planting densities (23, 37, and 51 plants m^–2^), with (52 μmol m^–2^ s^–1^) and without far-red radiation (FR) at 14 **(A)**, 21 **(B)**, and 28 **(C)** days after transplanting (DAT). Error bars indicate standard errors of means. For 14 DAT, a significant interaction between FR and planting density was observed (*P* = 0.027; *n* = 2). For 21 DAT, no significant interaction (*P* = 0.70) between effect of planting density (*P* = 0.59) and effect of FR (*P* = 0.26) was found. For 28 DAT, there was a significant interaction (*P* = 0.055; *n* = 3). Data are means of two (*n* = 2) or three blocks (*n* = 3) each with four–six replicate plants.

No difference of specific leaf area (SLA) among treatments was observed at 14 and 21 DAT (Figure 7). At 28 DAT, SLA was significantly affected by planting densities but not by FR (Figure 7). Similarly, the increment in SLA during the final cultivating week, from 21 to 28 DAT, was significantly different among planting densities and not affected by FR (not shown).

### Yield Component Analysis

**FIGURE 8:**
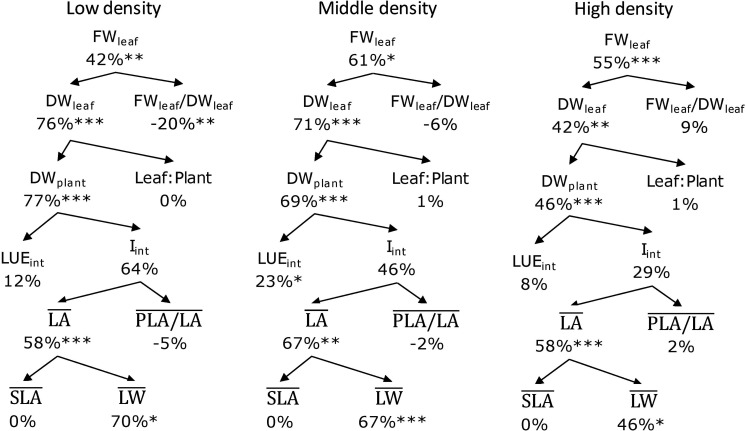
Effect of adding far-red radiation (FR) on top of red and blue at three planting densities. Percentages are the RB + FR increment on top of RB. Abbreviations within schemes are as follows: FW_leaf_, leaf fresh weight; DW_leaf_, leaf dry weight; FW_leaf_/DW_leaf_, leaf fresh/dry weight ratio; DW_plant_, plant total dry weight; Leaf:Plant, ratio of leaf dry weight in total plant; LUE_int_, intercepted light use efficiency; *I*_int_, canopy-intercepted photosynthetic photon flux density; $\bar{\text{LA}}$, plant leaf area; $\bar{\mathrm{PLA}/\mathrm{LA}}$, projected leaf area and leaf area ratio; $\bar{\text{SLA}}$, specific leaf area; $\bar{\text{LW}}$, leaf weight. The $\bar{\text{LA}}$, $\bar{\mathrm{PLA}/\mathrm{LA}}$, $\bar{\text{SLA}}$, and $\bar{\text{LW}}$ are all averaged values over 14, 21, and 28 days after transplanting (DAT) representing cumulative values during the whole cultivating period (0–28 DAT). ^∗^*P* < 0.10, ^∗∗^*P* < 0.05, and ^∗∗∗^*P* < 0.01. Data are means of two (*n* = 2) or three blocks (*n* = 3) each with four–six replicate plants.

FR increased leaf fresh weight (FW_leaf_) for all planting densities by 42–61%. This was the result of increased leaf dry weight (DW_leaf_) and not a higher fresh/dry weight ratio (FW_leaf_/DW_leaf_); this ratio actually was lower at RB + FR at the low planting density. FR increased DW_plant_ by 46–77%, which was mainly due to a higher canopy-intercepted PPFD (*I*_int_), which increased by 29–64%, and to a smaller extent (8–23%) by higher intercepted light use efficiency (LUE_int_). The higher *I*_int_ was caused by an increased average leaf area (LA_plant_) by 58–67%, rather than plant openness (PLA/LA), which varied little between treatments with and without FR. FR increased overall biomass partitioning to leaf (Figure 4), which led to a higher leaf area with a relative constant specific leaf area (SLA). The overall reasoning based on the component analysis (Figure 8) was supported by the correlation analysis (Supplementary Table S2).

## Discussion

Higher efficiencies of the photochemistry of photosystem II (PSII) and I (PSI), which are maximumly excited at 680 and 700 nm, respectively, contribute to a higher photosynthesis rate (Baker, 2008). Due to Emerson enhancement effect, the PSII efficiency might be increased by adding FR, hence the net photosynthetic rate increases in short term (Emerson et al., 1957). Zou et al. (2019) observed a 7–10% immediate increment in net photosynthesis rate by adding FR on top of plants acclimated to environments with and without FR. However, due to a lower chlorophyll and total nitrogen content as well as lower leaf absorbance, FR-acclimated plant’s photosynthetic capacity decreased in the long-term (Ji et al., 2019; Zou et al., 2019). In the present study, we did not find an effect of FR on leaf net photosynthesis rate, 9.8 ± 0.2 μmol (CO_2_) m^–2^ s^–1^in average (Supplementary Table S3), when measured under the light conditions. The plants were grown at 20 DAT, which resulted in similar results as reported by Ji et al. (2019) and Zhang et al. (2019) in tomato. There is a possible cancelling out of a positive instantaneous effect on net photosynthesis rate (Emerson enhancement effect) and lowered chlorophyll content per unit leaf area by FR-enriched environment acclimation. Plants acclimate to the growing light environment by adapting photochemistry system under RB or RB + FR conditions to utilize absorbed photons efficiently (Walters, 2005; Zhen et al., 2019). As shown by Ji et al. (2019) and Kalaitzoglou et al. (2019) in tomato, on the long run, the effect of FR on plant growth via affecting leaf photosynthesis rate is limited. The significantly higher biomass production is rather due to a substantial increment of photosynthetic leaf area by adding FR (Figure 3).

Adding FR on top of red and blue increased plant fresh weight significantly at all three planting densities (Figures 3, 8). This resulted from a higher total plant dry weight (DW_plant_) as well as leaf dry weight (DW_leaf_) in agreement with previous studies (Meng et al., 2019; Zou et al., 2019). The significantly higher DW_leaf_ when FR was added was due to a substantially higher leaf area (Figure 3). Several papers (Franklin, 2008; Vos et al., 2010; Bongers et al., 2014) have reviewed the effect of lowered R/FR, which typically happens in vegetation proximity where red (R) photons were mainly absorbed and thus the ratio between R and FR decreases. Lowering R/FR may result in more expansion of leaf area but not increases in the leaf number (Supplementary Table S3). In our experiment, an increase in expansion of leaf area by FR resulted in a cumulative advantage in intercepting a much higher fraction of incident light, resulting in an increase in plant dry weight (Figures 3, 8) at all planting densities. The more rapid expansion of leaf area resulted in a larger fraction of floor cover and consequently a higher light interception for the RB + FR treatment (Figure 5). The incident light use efficiency (LUE_inc_: plant dry weight per unit of cumulative incident PPFD) was consequently increased by adding FR. Radiation use efficiency (RUE: plant dry weight per unit of cumulative incident PFD) was also improved due to the strong increase in radiation interception by the enhanced leaf area expansion, which is in agreement with the lettuce experiment of Zou et al. (2019). The intercepted light use efficiency (LUE_int_: plant dry weight per unit of canopy-intercepted PPFD) was significantly increased by FR but no planting density effect was observed, which was in line with the results of instantaneous net photosynthesis rate increase when adding FR on top of R and B (Zou et al., 2019). The effects of FR on plant dry weight were stronger at low planting density, which could be explained by the fact that at low planting density, the light interception is lower and therefore an increase in light interception will have a larger effect on plant growth. Surprisingly, this stronger effect at low planting density was not observed for fresh weight, as the ratio of fresh to dry weight was strongly reduced at low planting density. Unexpectedly, this ratio did not decrease by FR at high planting density. In basil plants, the fresh-to-dry ratio was also reduced by FR (Larsen et al., 2020). A higher fraction of biomass partitioning to the shoot is one of the effect of lowered R/FR (Vos et al., 2010; Bongers et al., 2014). In case of lettuce, which has only a very small stem, it is the leaf that benefits from this. The increase of leaf/root ratio under FR suggests the relative sink strength of leaves had increased compared to that of the root (Marcelis, 1996; Heuvelink, 1997). Specific leaf area (SLA) has often been found to increase by additional FR or a lowered R/FR, which normally happens in vegetation proximity (Ballaré and Pierik, 2017), but not in the current research (Figure 8). Therefore, a higher fraction of biomass partitioned to the leaf resulted in a larger leaf area.

Considering that FR resulted in a higher biomass partitioning to the shoot, a higher leaf area, and improved light interception, the data suggest that adding FR on top of PAR is likely more efficient for dry weight production than adding same intensity PAR. The radiation use efficiency was indeed higher for plants grown with additional FR compared to no FR. It would be worthwhile to grow plants with and without FR, but with same total radiation, in order to test if the addition of FR is more efficient in promoting growth than addition of extra PAR.

## Conclusion

Our results demonstrate that adding FR on top of red and blue light increased lettuce fresh and dry weight significantly at three planting densities. The effects on dry weight were strongest at low planting density. The increased plant growth by adding FR was caused by a higher light interception by an enlarged leaf area resulting from a higher biomass partitioning to shoot, rather than from a higher leaf photosynthesis rate or specific leaf area. FR increased incident light use efficiency and radiation use efficiency, while it increased intercepted light use efficiency to a lesser extent.

## Data Availability Statement

The original contributions presented in the study are included in the article/Supplementary Material, further inquiries can be directed to the corresponding author/s.

## Author Contributions

WJ, EH, and LM conceived and designed the experiments. WJ and JU performed the experiments. WJ and EH performed statistical analysis. WJ wrote the manuscript. EH and LM revised and edited the manuscript. All authors contributed to manuscript revision and read and approved the submitted version.

## Conflict of Interest

The authors declare that this study received funding from Priva, De Lier, Netherlands. The funder was not involved in the study design, collection, analysis, interpretation of data, the writing of this article or the decision to submit it for publication. The authors declare that the research was conducted in the absence of any commercial or financial relationships that could be construed as a potential conflict of interest.
