# Supplementary material for: Adding Far-Red to Red-Blue Light-Emitting Diode Light Promotes Yield of Lettuce at Different Planting Densities
Source: Front Plant Sci. 2021 Jan 15;11:609977. doi: 10.3389/fpls.2020.609977 (PMC7845693; doi:10.3389/fpls.2020.609977)
Supplement: Supplementary file 1 [file Table_1.DOCX]

**Supplementary Table S1.** Treatments distribution in the climate chamber (Droevendaalsesteeg 1, 6708 PB, Radix, Wageningen University & Research, Wageningen, the Netherlands) in 3 replicates.

| Replicate | Side | Upper layer | Lower layer |
| --- | --- | --- | --- |
| 1 | South | RB+FR High and Middle density | RB+FR Low density |
|  | North | RB Low density | RB High and Middle density |
| 2 | South | RB High and Middle density | RB Low density |
|  | North | RB+FR High and Middle density | RB+FR Low density |
| 3 | South | RB Low density | RB High and Middle density |
|  | North | RB+FR High and Middle density | RB+FR Low density |

**Supplementary Table S2.** Correlation analysis output for all components in component analysis (Fig. 3, 8) and some LUE_inc_, LWR, Pn and RUE. FW_leaf_: leaf fresh weight; DW_leaf_: leaf dry weight; FW_leaf_/DW_leaf_: fresh - dry leaf weight ratio; DW_plant_: total plant dry weight; Leaf:Plant: fraction of biomass partitioning to leaf; I_int_: canopy intercepted photosynthetic photon flux density (PPFD) which is the cumulative PPFD interception during the whole cultivating period (0-28 DAT); LUE_int_: the dry weight production per unit of canopy intercepted PPFD; RUE: plant total dry weight per cumulative incident radiation including PAR and FR; LUE_inc_: plant total dry weight per unit of cumulative incident PAR; $\bar{\mathrm{LA}}$: leaf area per plant; $\bar{PLA/LA}$: plant openness defined as the ratio between projected leaf area and leaf area; $\bar{\mathrm{LW}}$: leaf dry weight; $\bar{\mathrm{SLA}}$: specific leaf area. The $\bar{\mathrm{LA}}$, $\bar{PLA/LA}$, $\bar{\mathrm{SLA}}$ and $\bar{\mathrm{LW}}$ were averaged over 14, 21 and 28 DAT representing the average levels of all parameters during the whole cultivating period (0-28 DAT) (n=3 for components numbered 1-11 and n=2 for components numbered 12-15). Correlation level in bold indicates a significant correlation. *P<0.10 and **P<0.05.

| DW_leaf_ | 1 | - |  |  |  | |  | |  | |  | | |  | | |  | | |  | | |  | | |  | | |  | | |  | | | |  | | | |  |  |  |
| --- | --- | --- | --- | --- | --- | --- | --- | --- | --- | --- | --- | --- | --- | --- | --- | --- | --- | --- | --- | --- | --- | --- | --- | --- | --- | --- | --- | --- | --- | --- | --- | --- | --- | --- | --- | --- | --- | --- | --- | --- | --- | --- |
| DW_plant_ | 2 | **1.00**** | - |  |  | |  | |  | |  | | |  | | |  | | |  | | |  | | |  | | |  | | |  | | | |  | | | |  |  |  |
| FW_leaf__DW_leaf_ | 3 | 0.16 | 0.18 | - | |  | |  | |  | |  | | |  | | |  | | |  | | |  | | |  | | |  | | |  | | | |  | | | |  |  |
| $\bar{\mathrm{LA}}$ | 4 | **0.52**** | **0.54**** | -0.33 | | - | |  | |  | |  | | |  | | |  | | |  | | |  | | |  | | |  | | |  | | | |  | | | |  |  |
| Leaf_Plant | 5 | 0.17 | 0.09 | -0.21 | | -0.16 | | - | |  | |  | | |  | | |  | | |  | | |  | | |  | | |  | | |  | | | |  | | | |  |  |
| Leaf_root | 6 | -0.03 | -0.02 | 0.10 | | -0.24 | | -0.07 | | - | | |  | | |  | | |  | | |  | | |  | | |  | | |  | | |  | | | |  | | | |  |
| Pn | 7 | 0.24 | 0.22 | -0.34 | | 0.35 | | 0.29 | | -0.19 | | | - | | |  | | |  | | |  | | |  | | |  | | |  | | |  | | | |  | | | |  |
| RUE | 8 | **0.96**** | **0.96**** | 0.25 | | 0.30 | | 0.16 | | 0.07 | | | 0.12 | | | - | | |  | | |  | | |  | | |  | | |  | | |  | | | |  | | | |  |
| $\bar{\mathrm{SLA}}$ | 9 | **0.46*** | **0.45*** | 0.07 | | 0.04 | | 0.26 | | -0.15 | | | 0.19 | | | **0.49**** | | | - | | |  | | |  | | |  | | |  | | |  | | | |  | | | |  |
| LUE_inc_ | 10 | **1.00**** | **1.00**** | 0.18 | | **0.54**** | | 0.08 | | -0.03 | | | 0.21 | | | **0.96**** | | | **0.45*** | | | - | | |  | | |  | | |  | | |  | | | |  | | | |  |
| FW_leaf_ | 11 | **0.93**** | **0.94**** | **0.49**** | | 0.36 | | 0.07 | | -0.01 | | | 0.05 | | | **0.93**** | | | **0.46*** | | | **0.94**** | | | - | | |  | | |  | | |  | | | |  | | | |  |
| I_int_ | 12 | **0.86**** | **0.86**** | -0.07 | | 0.43 | | 0.14 | | 0.33 | | | 0.11 | | | **0.86**** | | | **0.70**** | | | **0.85**** | | | **0.70**** | | | - | | |  | | |  | | | |  | | | |  |
| LUE_int_ | 13 | 0.31 | 0.33 | 0.49 | | 0.38 | | -0.11 | | **-0.69**** | | | -0.06 | | | 0.23 | | | 0.18 | | | 0.35 | | | 0.48 | | | -0.19 | | | - | | | |  | | | |  | | | |
| $\bar{PLA/LA}$ | 14 | 0.08 | 0.07 | **-0.59**** | | 0.00 | | 0.10 | | **0.53*** | | | 0.19 | | | 0.09 | | | 0.15 | | | 0.04 | | | -0.18 | | | **0.52*** | | | **-0.85**** | | | | - | | | |  | | | |
| $\bar{\mathrm{LW}}$ | 15 | **0.99**** | **0.99**** | 0.25 | | **0.53*** | | 0.14 | | 0.01 | | | 0.03 | | | **0.95**** | | | **0.81**** | | | **0.99**** | | | **0.95**** | | | **0.85**** | | | 0.33 | | | | 0.04 | | | | - | | | |
|  |  | 1 | 2 | 3 | | 4 | | 5 | | 6 | | | 7 | | | 8 | | | 9 | | | 10 | | | 11 | | | 12 | | | 13 | | | | 14 | | | | 15 | | | |

**Supplementary Table S3.** Actual values the plant growth components shown in Figure xx, averaged over 3 blocks. Values followed by different letters are significantly different according to Student's protected LSD test at P=0.05. When interaction between FR treatment and plant density was significant, letters refer to comparison of the six interaction means. When interaction was not significant, letters refer to comparing the 3 averages for plant density and the 2 averages for FR treatment separately.

| Leaf Fresh Weight (g·plant^-1^) | Light treatment | |  |
| --- | --- | --- | --- |
| Planting density | RB | RB+FR | Mean |
| Low | 40.7 | 57.6 | 49.1a |
| Middle | 36.6 | 59.0 | 47.8a |
| High | 35.5 | 55.0 | 45.2a |
| Mean | 37.6a | 57.3b |  |
| Leaf Dry Weight (g·plant^-1^) | Light treatment | |  |
| Planting density | RB | RB+FR | Mean |
| Low | 2.66a | 4.70d | 3.68 |
| Middle | 2.43a | 4.16c | 3.30 |
| High | 2.40a | 3.40b | 2.90 |
| Mean | 2.50 | 4.08 |  |
| Leaf Fresh Weight/Leaf Dry Weight (-) | Light treatment | |  |
| Planting density | RB | RB+FR | Mean |
| Low | 15.3bc | 12.3a | 13.8 |
| Middle | 15.0bc | 14.1b | 14.6 |
| High | 14.8bc | 16.1c | 15.5 |
| Mean | 15.1 | 14.2 |  |
| Plant Dry Weight (g·plant^-1^) | Light treatment | |  |
| Planting density | RB | RB+FR | Mean |
| Low | 3.14a | 5.54d | 4.34 |
| Middle | 2.91a | 4.91c | 3.91 |
| High | 2.73a | 3.98b | 3.36 |
| Mean | 2.92 | 4.81 |  |
| Leaf Dry Weight/Plant Dry Weight (-) | Light treatment | |  |
| Planting density | RB | RB+FR | Mean |
| Low | 0.85 | 0.85 | 0.85a |
| Middle | 0.84 | 0.85 | 0.84a |
| High | 0.88 | 0.85 | 0.86a |
| Mean | 0.85a | 0.85a |  |
| Intercepted light use efficiency (g·mol^-1^) | Light treatment | |  |
| Planting density | RB | RB+FR | Mean |
| Low | 1.11 | 1.25 | 1.18a |
| Middle | 1.03 | 1.27 | 1.15a |
| High | 1.14 | 1.23 | 1.19a |
| Mean | 1.10a | 1.25b |  |
| Canopy intercepted PPFD at 14 DAT (mol·m^-2^) | Light treatment | |  |
| Planting density | RB | RB+FR | Mean |
| Low | 6.68 | 7.76 | 7.22a |
| Middle | 11.63 | 16.39 | 14.01b |
| High | 14.94 | 21.06 | 18.00c |
| Mean | 11.08a | 15.07a |  |
| Canopy intercepted PPFD at 21 DAT (mol·m^-2^) | Light treatment | |  |
| Planting density | RB | RB+FR | Mean |
| Low | 24.9 | 37.7 | 31.3a |
| Middle | 46.2 | 65.9 | 56.1b |
| High | 51.1 | 77.9 | 64.5c |
| Mean | 40.8a | 60.5b |  |
| Canopy intercepted PPFD at 28 DAT (mol·m^-2^) | Light treatment | |  |
| Planting density | RB | RB+FR | Mean |
| Low | 67.6 | 108.0 | 87.8a |
| Middle | 116.1 | 155.1 | 135.6b |
| High | 130.8 | 171.3 | 151.1c |
| Mean | 104.8a | 144.8b |  |
| Leaf area (cm^2^·plant^-1^) | Light treatment | |  |
| Planting density | RB | RB+FR | Mean |
| Low | 177.5 | 280.6 | 229.1a |
| Middle | 169.2 | 283.2 | 226.2b |
| High | 157.8 | 249.6 | 203.7c |
| Mean | 168.2a | 271.1b |  |
| Projected leaf area/Leaf area (-) | Light treatment | |  |
| Planting density | RB | RB+FR | Mean |
| Low | 0.48 | 0.46 | 0.47a |
| Middle | 0.53 | 0.52 | 0.53a |
| High | 0.49 | 0.50 | 0.49a |
| Mean | 0.50a | 0.49a |  |
| Specific leaf area (cm^2^·g^-1^) | Light treatment | |  |
| Planting density | RB | RB+FR | Mean |
| Low | 294 | 295 | 294a |
| Middle | 305 | 323 | 314b |
| High | 333 | 336 | 334c |
| Mean | 311a | 318a |  |
| Leaf weight (g·plant^-1^) | Light treatment | |  |
| Planting density | RB | RB+FR | Mean |
| Low | 2.67a | 4.69d | 3.68 |
| Middle | 2.43a | 4.16c | 3.29 |
| High | 2.40a | 3.34b | 2.90 |
| Mean | 2.50 | 4.08 |  |
| Instantaneous net photosynthesis rate  (µmol(CO_2_)·m^2^·s^-1^) | Light treatment | |  |
| Planting density | RB | RB+FR | Mean |
| Low | 9.7 | 9.9 | 9.8a |
| Middle | 9.5 | 10.0 | 9.8a |
| High | 9.7 | 9.9 | 9.8a |
| Mean | 9.6a | 9.9a |  |
| Leaf number (leaves·plant^-1^) | Light treatment | |  |
| Planting density | RB | RB+FR | Mean |
| Low | 18.8 | 20.5 | 19.7a |
| Middle | 19.6 | 20.5 | 20.1a |
| High | 18.1 | 18.5 | 18.3a |
| Mean | 18.9a | 19.8a |  |
| Incremental SLA from 14 to 21 DAT  (DW_leaf21_ – DW_leaf14_)/(LA_21_ – LA_14_) | Light treatment | |  |
| Planting density | RB | RB+FR | Mean |
| Low | 271 | 298 | 284a |
| Middle | 262 | 341 | 301a |
| High | 282 | 312 | 297a |
| Mean | 272a | 317a |  |
| Incremental SLA from 21 to 28 DAT  (DW_leaf28_ – DW_leaf21_)/(LA_28_ – LA_21_) | Light treatment | |  |
| Planting density | RB | RB+FR | Mean |
| Low | 242 | 210 | 226a |
| Middle | 257 | 249 | 253b |
| High | 262 | 293 | 277c |
| Mean | 254a | 251a |  |
| Plant openness (PLA/LA) averaged 14, 21 and 28 DAT | Light treatment | |  |
| Planting density | RB | RB+FR | Mean |
| Low | 0.48 | 0.46 | 0.47a |
| Middle | 0.53 | 0.52 | 0.53a |
| High | 0.49 | 0.50 | 0.49a |
| Mean | 0.50a | 0.49a |  |
